# Optimization of dewatering process of concentrate pressure filtering by support vector regression

**DOI:** 10.1038/s41598-022-11259-9

**Published:** 2022-05-17

**Authors:** Huizhong Liu, Keshun You

**Affiliations:** Present Address: School of Mechanical and Electrical Engineering, Jiangxi University of Science and Technology, Ganzhou, 341000 China

**Keywords:** Engineering, Mechanical engineering

## Abstract

This work studies the mechanism and optimization methods of the filter press dehydration process to better improve the efficiency of the concentrate filter press dehydration operation. Machine learning (ML) models of radial basis function (RBF)–OLS, RBF-generalized regression neural network, and support vector regression (SVR) are constructed, and laboratory and industrial simulations are performed separately, finally, optimization methods for the filtration dewatering process are designed and applied. In laboratory, all the machine learning models have obvious mistakes, but it can be seen that SVR has the best simulation effect. In order to achieve the optimization of the entire filtration and dewatering process, we obtained enough data from the industrial filtration and dewatering system, and in the industrial simulation results all the machine learning models performed considerably, SVR achieves the best accuracy in industrial simulation, and the simulated mean relative error of moisture and processing capacity are 1.57% and 3.81%, the model was tested with newly collected industrial data to verify the credibility. The optimal simulation results are obtained by optimization method based on control variables. Results show that the ML method of SVR and optimization methods of control variables applied to the industry not only can save energy consumption and cost but also can improves the efficiency of filter press operation fundamentally, which will provide some options for intelligent dewatering process and other industrial production optimization.

## Introduction

The gradual exhaustion of easy-beneficiation mineral resources has increased the amount of complex and low-grade ore and has made the grain size of grinding finer. Thus, the dewatering and filtration of concentrate become increasingly difficult. A high-efficiency pressure filter has been developed and gradually applied to the Filter press dehydration of concentrate. Because the automatic control technology in pressure filter, this type of high-efficiency pressure filter is generally called an automatic pressure filter^1^. The forced dehydration process of “mechanical pressing” and “air drying” on the basis of the “feeding pressure” of the conventional filter press is applied in automatic pressure filter^2^. Thus, it not only can obtain a filter cake with lower moisture but also has a higher operating efficiency^3^.

Many kinds of automatic filter presses have been successfully used in the mineral processing industry, such as the Larox-PF automatic filter press developed by Larox in Finland^4,5^ and the BPF automatic filter press in China^6^. The dehydration process of the automatic pressure filter is relatively complicated, and the stability of the index and the efficiency of the dehydration process are affected by the reasonableness of the control parameter setting of the dehydration process^7^. Thus, the optimization research of the filter press dehydration process control has received an increased attention^8^.

At present, there are many researches about optimization of dehydration, such as designing a dehydration circuit^9^, optimizing the filter medium^10,11^, driving the electric dehydration by pressure^12,13^. Although electrically driven dehydration is highly efficient, it is energy-consuming and low stability performance. And it is heavily limited by the research of materials science. However, it is a new idea to constructing coordinated optimization models by collecting production data to solve the problems encountered in the dehydration industry^14^. Some dehydration optimizations are used through chemical methods, dewatering aids, and flocculant to optimize the dehydration of mineral particles^15–18^, even through a combination of physical and chemical with double optimization methods to optimize dehydration^19,20^, which is undeniable that these are indeed some exciting findings, but from a macro point of view, they are all problem-oriented dehydration optimizations and the efficiency of the concentrate filter press dehydration operation is restricted. The leapfrog multi-parameter overall optimization is the realization of the common adaptive adjustment of the filter press dehydration system, which is an important step in the intelligentization of the dehydration system in the future.

Thus, the optimization method of dehydration process by ML (Machine learning) and data-driven has the best advantages and the most long-term significance. Experimental data are used^21^ to characterize industrial dehydration performance. A mechanism model of the filter press process is established^22^ to obtain the best operating control parameters of the filter press process for achieving process optimization. Industrial pressure filtration with process data is analyzed and modeled^23^. Although the application of ML to the optimization of the parameters of the dehydration process has been studied, there are relatively few optimized parameters in the system, and the data for modeling are all obtained at one time, which will eventually lead to no authenticity of the model because of no verification, more importantly, since these constructed machine learning models are not combined with corresponding optimization methods, continuous optimization cannot be performed, resulting in limited optimization results, which does not fundamentally improve the efficiency of the filter press dehydration process.

In the study, through a combination of laboratory and industrial dehydration experiments, multiple sets of industrial production data are used to establish ML models of pressure filter dehydration process control. Comparing the simulation and prediction accuracy of the three ML models of orthogonal least square (OLS) and generalized regression neural network (GRNN) with radial basis function (RBF) networks and support vector regression (SVR) shows that SVR has the highest accuracy. Simultaneously, we repeatedly obtain data from the industrial filter press dehydration system to construct a multi-parameter model and conduct model tests. We study an optimization method of control variables to obtain the optimal control parameters in the control model. This method aims to optimize another parameter while ensuring that one control parameter is qualified. In this way, the optimal control parameters in the industry are obtained. In the end, we verified the rationality of our optimization process and method, and the continuous optimization of the industrial filter press dehydration process is realized.

## Design of optimization methods for dehydration processes

A complete working process of the automatic pressure filter includes closing the filter plate-feeding and filtering, mechanical pressing, air-drying, opening the filter plate, unloading the cake, and cleaning the filter cloth. Processing capacity and moisture are mainly included in dehydration index, which is determined by the three main dehydration processes of “feeding filter press”, “mechanical pressing”, and “air drying”. Only a good understanding of the filter press dehydration process and the purpose of optimization can better put forward the optimization method of control parameters of the actual filter press dehydration process. Therefore, in this chapter, we first introduce the three-stage process of filter press dehydration. Then, we propose an optimization method based on the control variable according to the purpose of process optimization.

### Feeding filter press process

The feeding filter press process is the process of pressing the slurry into the filter chamber. This process starts the filtration operation when the slurry is hydraulically inserted into the filter chamber. It is a process of filter cake filtration and follows the following basic filtration equation proposed in Eq. (1)^24^.

1 $$Q = \frac{dV}{{dt}} = K\frac{A\Delta P}{{\mu L}},$$

where *Q* is the flow rate of the filtrate; *A* is the filtration area; *t* is the filtration time; *V* is the volume of the filtrate accumulated in time t; *L* is the thickness of the filter layer; *K* is the permeability coefficient of the filter layer; Δ*P* is the cross-filtration layer of the pressure drop (the driving force of filtration); *μ* is the viscosity of the filtrate.

In the case of constant feed concentration and pressure, the length of feed time directly determines the thickness of the filter cake. With the extension of the feeding time, the filter cake gradually thickens, but the thickening speed is rapidly decreasing. Prolonging the feeding time can increase the thickness of the filter cake and the output of the filter cake per press filter cycle. However, if the feeding time is prolonged, then it will significantly increase the filter press cycle, which in turn will reduce the cake output per unit time. Therefore, “feeding time” is an important factor that affects the dehydration index and efficiency. According to the nature of the material (filter cake thickness), obtaining a reasonable feeding time is the goal of this process optimization.

### Press dehydration process

**Figure 1:**
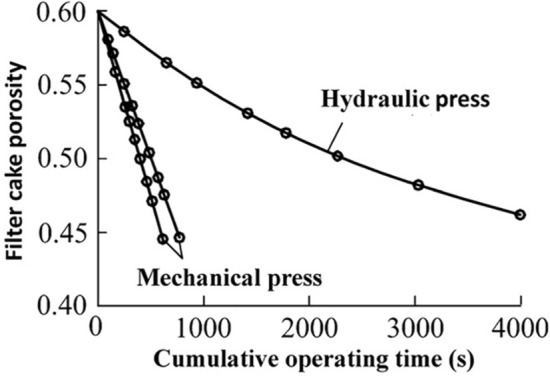
Mechanical and hydraulic pressing test curve.

Press dehydration can be conducted in many ways, and the most widely used are mainly two methods: one is mechanical press dehydration with a pressing mechanism, and the other is hydraulic press dehydration. The comparison of the effects of the two pressing methods is shown in Fig. 1 according to existing research^25^. As observed, the mechanical pressing curve is obviously steeper, which means that the time to reach the same filter cake porosity after the filter cake is mechanically pressed is significantly shorter. Mechanical pressing can significantly shorten the dehydration time of the filter cake.

After the “feeding filter press” is completed, the “mechanical press” stage begins. The automatic pressure filter uses a diaphragm press, which is a type of mechanical press. If the pressing time is insufficient, the water in the pores of the filter cake is not fully squeezed out, and the pressing is stopped, then the final moisture of the filter cake will increase, which will affect the efficiency of pressing. On the contrary, after a certain period of time of filter cake pressing, the porosity of the filter cake no longer decreases, and the water content does not decrease anymore. If the pressing time continues to be extended, then the working cycle of the filter press will be prolonged and the pressing power will be consumed.

### Air-drying process

**Figure 2:**
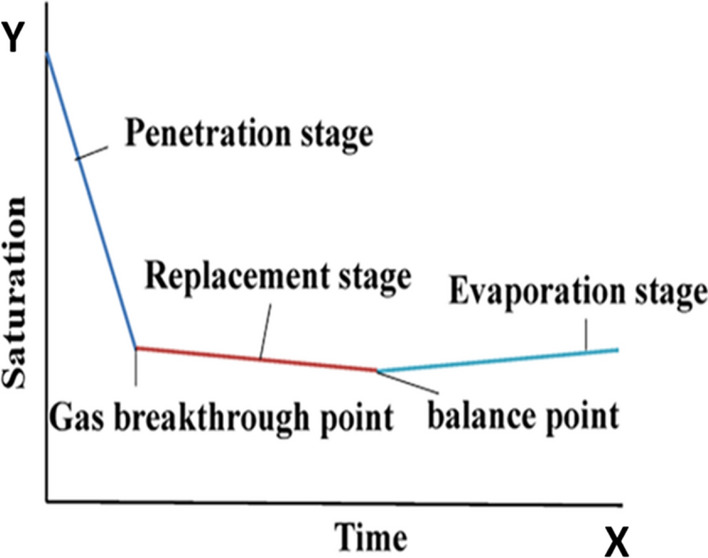
Three stages of air drying and dehydration.

After the process of pressing is completed, some moisture still remains in the pores of the filter cake. At this time, if compressed air is passed into the filter chamber, through the filter cake, and the residual moisture in the filter cake is further removed, then air drying and dehydration can be realized. According to existing research^26^, air drying can be divided into three stages: penetration, replacement, and evaporation stages. The effect of each stage is shown in Fig. 2.

Figure 2 shows that the liquid discharge is the most in the penetration stage, and then, the displacement and evaporation stages begin. After the penetration phase is over, the saturation of the filter cake drops slightly, and the moisture of the filter cake is close to the final moisture. If blowing and drying are continued, then only the consumption of compressed air will be increased. Therefore, under certain air-drying pressure conditions, reasonable control of the air-drying time and timely termination of air-drying after the completion of the penetration stage are the goals of air-drying process optimization.

### Method of filter press dehydration optimization

For a filter press dehydration system of fixed concentrate, the “feed pressure” is related to the feed pump and is relatively fixed, thus, it will not be considered. The size of the concentrate slurry is relatively fixed, and the viscosity is directly related to the concentration. Therefore, the filter press optimization process considers the two conditions of “feeding concentration” and “squeezing pressure”. “Feeding time”, “press room”, and “air-drying time” are also the main optimization parameters of the filter press process^27^.

The entire dehydration process is controlled by the automatic control system of the filter press. On the one hand, the automatic control system of the filter press realizes the precise control of the mechanical action of the filter press itself. On the other hand, it realizes the program control and auxiliary dewatering process of “feeding, pressing, air-drying, cake unloading, and cloth washing”. It also adjusts the control parameters.

During the operation of the equipment, the operator needs to modify the main control parameters in time through the man–machine interface when slurry conditions such as “feeding concentration” or filtration index requirements change to ensure the filter press dehydration index and work efficiency. However, the current setting and modification of the control parameters of the filter press dehydration process are mainly conducted by the operators on the basis of their own experience and knowledge. The difference in experiences of different operators causes fluctuations in the index and efficiency of the filter press dehydration operation. Obtaining the optimal value of the main control parameters of the filter press dehydration process is the key to ensuring the efficient operation of the dehydration operation. Obviously, the optimal value cannot be obtained by relying only on the experience of the operator. Optimal parameters can be predicted by ML^28,29^. Therefore, we propose a precise ML model of filter press dehydration process that uses existing data samples. Then, we use the results of the ML model prediction to design a reliable optimization method for obtaining the optimal value of the control parameters. This way achieves the control optimization of filter press dehydration process. Through the study of the actual situation, we propose the following reasonable optimization principle (optimization methods based on the principle of controlled variables):

1. Under the premise of ensuring that the filter cake with qualified water content is obtained, the optimized control of parameters is used to obtain the maximum processing capacity per unit filter area.
2. Under the premise of ensuring the processing capacity per unit filter area, the filter cake with the lowest moisture can be obtained through optimized control of parameters.

**Figure 3:**
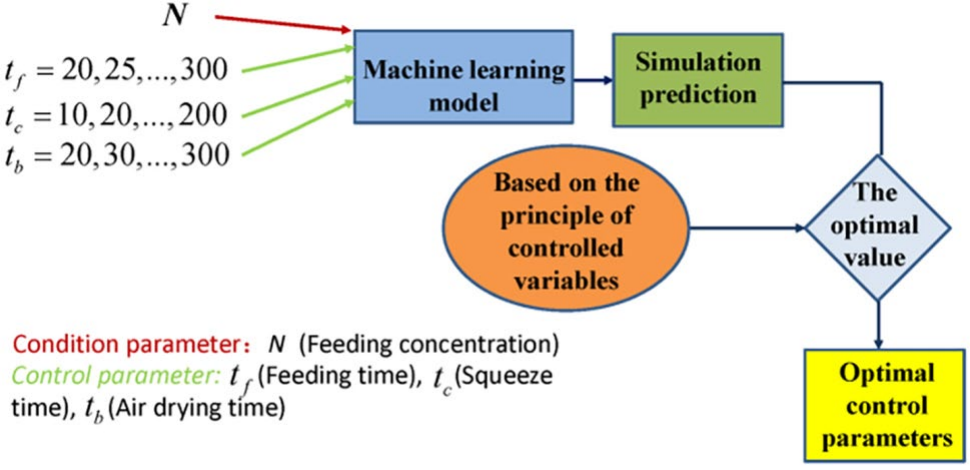
Optimization process of filter press dehydration.

As shown in Fig. 3, the principle of this optimization method based on the controlled variable is as follows. Under certain external conditions, each control parameter is sequentially selected from small to large within the reasonable value range of each control parameter, and the value is cyclically selected at small intervals. The values obtained by the control parameters are arranged and combined to obtain a combination of control parameters under various conditions. For each combination, the established ML model is used to obtain simulation results, and the simulation results are sequentially compared according to different optimization principles and optimization goals to find the optimal. As a result, the control parameter group corresponding to the optimal result is the optimal control parameter.

## Optimal model of filter press dehydration process in laboratory

ML and artificial intelligence techniques increasingly promote the development of mineral processing^30–32^. According to the previously introduced optimization principle and process of filter press dehydration, we need to build an accurate ML model to achieve the optimization of the filter press dehydration process. For better industrial applications, this study uses the OLS method and the GRNN method based on RBF neural network and SVR to construct an optimal control model for the filter press dehydration process.

### RBF ML model

Artificial neural network is a complex network system composed of many interconnected neurons. Many kinds of neural network models are available. However, we use the RBF neural network model based on OLS and GRNN to establish a simulation model of the filter press dehydration process.

**Figure 4:**
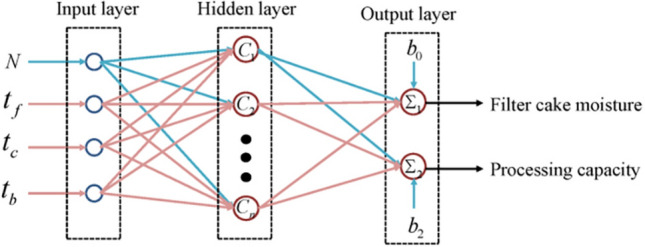
RBF neural network model structure of filter press dehydration process.

The RBF neural network model structure of the filter press dehydration process established by us is shown in Fig. 4. The RBF network is a two-layer network with only one hidden layer in addition to the input and output layers. The transfer function in the hidden layer is a Gaussian function of the local response, while the transfer function for other forward networks is generally a global response function. RBF needs more neurons to achieve the same function due to this difference. Thus, the RBF network cannot replace the standard forward network. However, the training time of RBF is shorter. It is optimal for function approximation and can approximate any continuous function with arbitrary precision. The approximation is more accurate when the hidden layer has more neurons.

**Figure 5:**
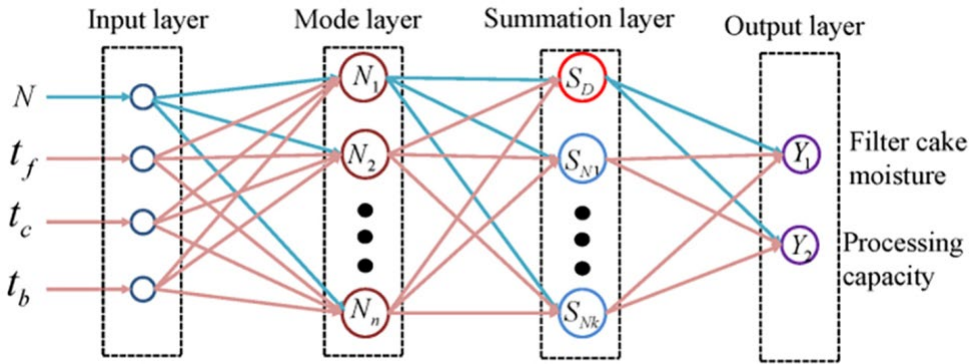
GRNN model structure of filter press dehydration process.

As shown in Fig. 5, GRNN is an improvement of RBF with similar structure^33,34^. The difference is that an extra layer of summation is considered, and the weight connection between the hidden and output layers (least square superposition of Gaussian weights) is removed. GRNN converges quickly because it has no model parameters to train. Based on the radial basis network, it also has good nonlinear approximation performance. However, each test sample of GRNN needs to be calculated with all training samples. Thus, its computational complexity is high. Furthermore, all training samples need to be stored. Accordingly, the space complexity is also high.

We also construct an RBF neural network model based on OLS. This model is established on the basis of constructing the RBF model structure through the least squares method. The optimization method of residual sum of squares is to optimize the parameters of the RBF network structure for minimizing the sum of squares of the difference between the regression function and the actual value. The OLS method regresses the predicted response variable through a series of predictor variables.

As shown in Eq. (2), the OLS linear regression aims to obtain model parameters by reducing the difference between the true value of the response variable and the predicted value. In this equation, *Yt* is called the dependent variable, *Xt* is called the independent variable, $\alpha$, $\beta$ are called regression coefficients, *t* = 1, 2, 3, 4, represents the number of observations, and $\mu_{t}$ represents the error.

2 $$Y_{t} = \alpha + \beta X_{t} + \mu_{t} .$$

As shown in Eq. (3), the OLS linear regression aims to best fit the curve to ensure that the sum of squares of the distances from each point to the straight line (i.e., the residual sum of squares, *RSS* for short) is the smallest.

3 $$RSS = \sum\limits_{t = 1}^{T} {\left( {y_{t} - \overset{\lower0.5em\hbox{$\smash{\scriptscriptstyle\frown}$}}{y}_{t} } \right)^{2} } = \sum\limits_{t = 1}^{T} {\left( {y_{t} - \overset{\lower0.5em\hbox{$\smash{\scriptscriptstyle\frown}$}}{\alpha } - \overset{\lower0.5em\hbox{$\smash{\scriptscriptstyle\frown}$}}{\beta } x_{t} } \right)^{2} } .$$

### Method of SVM and SVR

The support vector machine (SVM) was proposed by Vapnik et al. in the 1990s, and it is based on statistical learning theory^35,36^. SVM method is an ML method based on the VC dimension theory of statistical learning theory and the principle of structural risk minimization^36^. It is based on the complexity of the model of limited samples to get the best compromise between learning abilities for obtaining the best generalization ability. The number of samples should be high in neural network networks because they are based on the principle of empirical risk minimization. However, the SVM method is based on the principle of structural risk minimization. Thus, under the condition of small samples, the model established by the SVM method has better generalization and promotion performance^37^.

**Figure 6:**
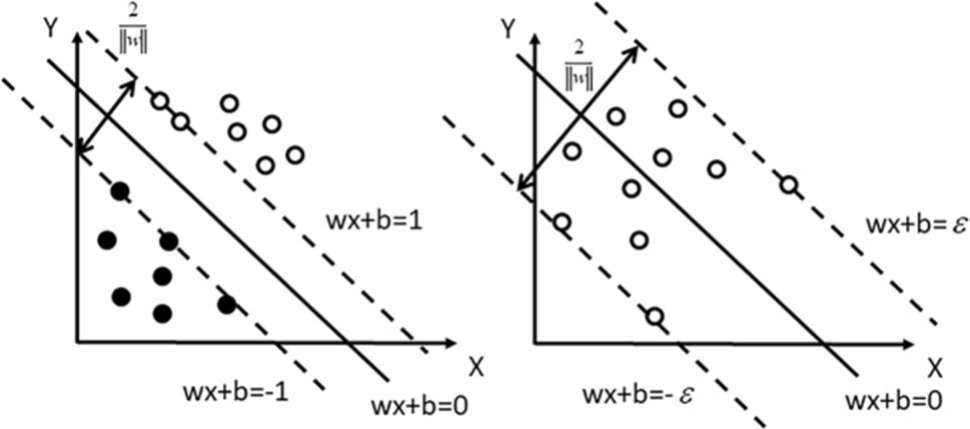
Method of SVM (on the left) and SVR (on the right).

SVM is developed from the optimal classification surface in the case of linear separability. The basic idea can be illustrated by the two-dimensional situation in Fig. 6. The solid and hollow dots represent two types of samples. *H* is the classification line. *H*_1_ and *H*_2_ are the lines that pass the closest samples to the classification line and are parallel to the classification line. The distance between them is called the classification interval (margin). The so-called optimal classification line requires that the classification line not only correctly separates the two categories but also maximizes the classification interval. This type of classification line equation can be defined as Eq. (4).

4 $$w \cdot x + b = 0.$$

When SVM normalizes the data, the linearly separable sample is required to satisfy Eq. (5).

5 $$y_{i} \left[ {\left( {w \cdot x} \right) + b} \right] - 1 \ge 0.$$

At this time, the classification interval is equal to 2/‖*w*‖, such that the maximum interval is equivalent to the minimum ‖*w*‖^2^. The classification surface that satisfies the condition (7) and minimizes the objective function as shown in Eq. (6). 2/‖*w*‖^2^ is called the optimal classification surface.

A plane with the farthest distance from the point on the boundary to the plane is found by classification, and $\varsigma$ of an insensitive loss function as the loss function is introduced in the SVR to minimize the distance from each point to the regression line, which is used for controlling the distance between the actual values and boundary values, and loss function is used for determining if the value of $w^{T} \Phi \left( {x_{i} } \right)$ is located in the range of y $\pm$ ε, then the calculation loss can be ignored^38,39^

6 $$\mathop {\min }\limits_{{}} \, \frac{1}{2}w^{T} w + C\sum\limits_{i = 1}^{n} {\left( {\varsigma_{i} + \varsigma_{i} *} \right)} ,$$

7 $${\text{Subject to}}\left\{ \begin{array}{*{20}l} y_{i} - w^{T} \Phi \left( {x_{i} } \right) - b \le \varepsilon + \varsigma_{i} \hfill \\ w^{T} \Phi \left( {x_{i} } \right) + b - y_{i} \le \varepsilon + \varsigma^{*}_{i} \hfill \\ \varsigma_{i} ,\varsigma^{*}_{i} \ge 0,i = 1,...,n \hfill \\ \end{array} \right..$$

### Laboratory simulation results of three ML models

After three ML models are constructed for the control and optimization of the filter press dehydration process, we conduct the filter press dehydration experiment in the laboratory. The data collected from laborator dehydration system is also used to explore the construction method of the filter press dehydration process simulation model.

**Figure 7:**
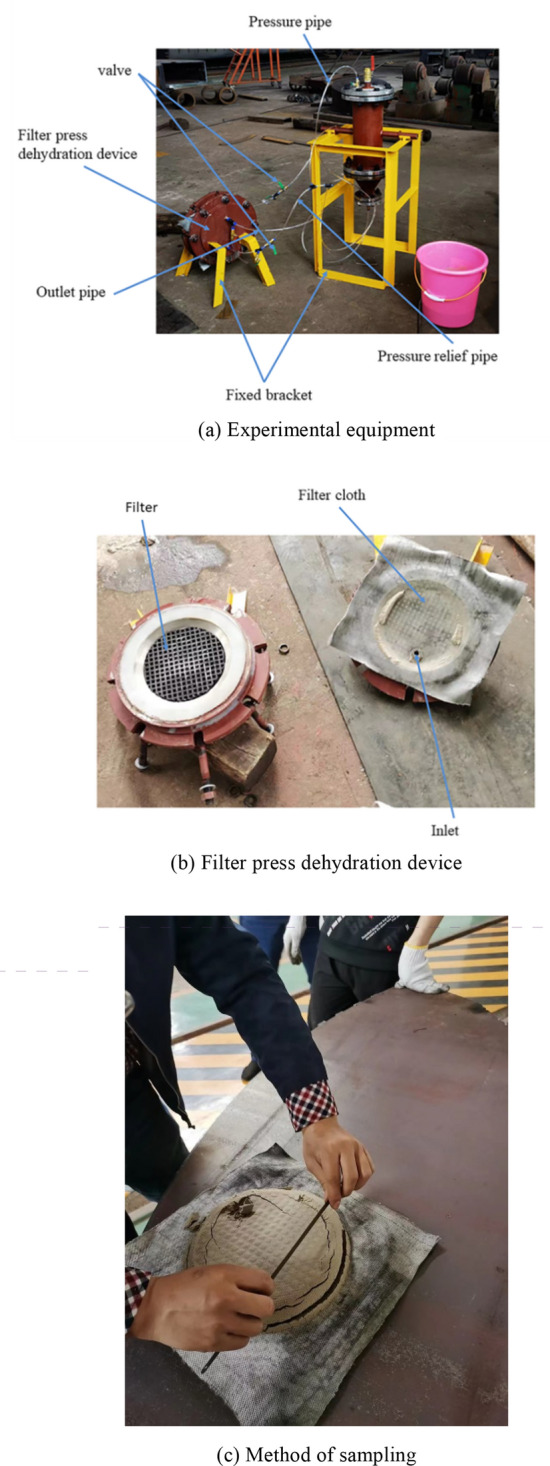
Experiment procedure.

Some rare earth ore are configured with concentration of 30%, and particle size of 0.02–0.07 mm, experiment procedure is shown in Fig. 7 and the self-developed micro-automatic filter press is used as the experimental equipment. The condition and control parameters are adjusted separately to conduct the filter press dehydration test of the material. After every test, the filter cake is sieved and sampled, and then dried after grinding. The area of the filter cake and the mass of the sample before and after drying are calculated, and the moisture and processing capacity data of each sample are calculated in turn. Before obtaining the data, we will change and adjust the input variables according to the actual situation, so that the filter cake moisture and processing capacity will meet the actual requirements of the industry before sampling. The normal fluctuation ranges of input variables, output filter cake moisture and unit area processing capacity are shown in Table 1.

**Table 1:** The ranges of the variables studied.

Feeding concentration/%	Feeding time/s	Squeezing time/s	Air-drying time/s	Cake moisture/%	Processing capacity (kg/m^2^/h)
20–65	10–120	10–180	30–80	< 20	> 70

**Figure 8:**
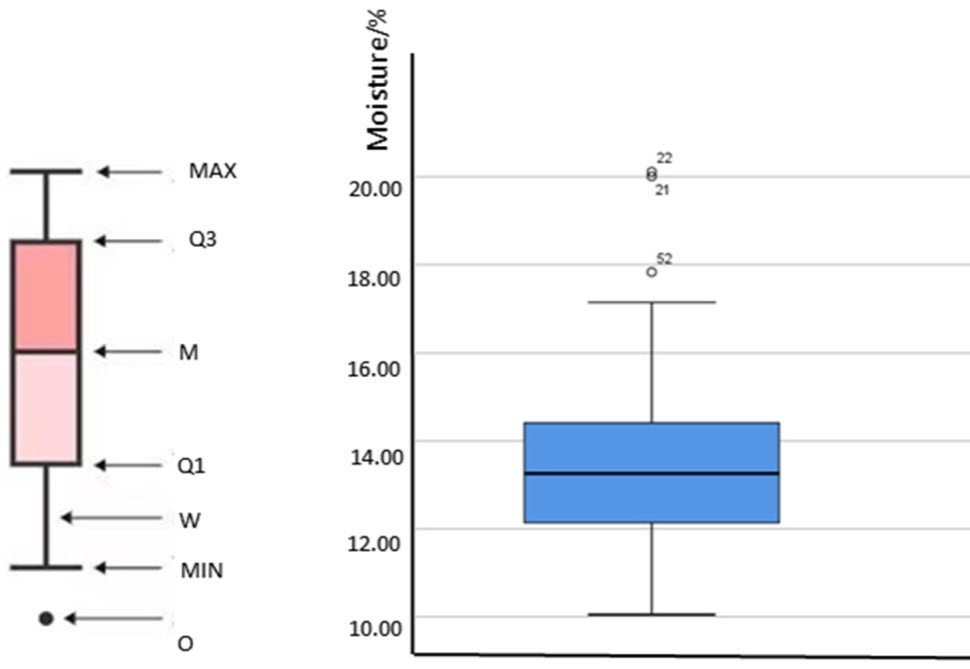
Abnormal data analysis.

A total of 163 sets of tests are conducted, and data are collected. As shown in Fig. 8, In order to analyze whether the acquired data is abnormal, we adopted the box diagram analysis method, and found that the most reasonable value of the filter cake moisture is between 12.1 and 14.5%. Where *MAX*, *MIN* are the limit values of moisture, *M* is the median of all moisture value data, Q1 is the first quantile, Q3 is the tertile, *W* is the whisker value, and *O* is the abnormal data. It can be seen from the box plot that there are three sets of data outside the box, which may be abnormal data, but considering that one of the sets of data meets the normal range of the research variables shown in Table 1.

Just Excluding 2 sets of abnormal data, 100 sets of the remaining 161 sets of data are used as training samples, and the remaining 61 sets are used as test samples to verify the simulation accuracy of the built model. “Feeding concentration”, “feeding time”, “squeezing time”, and “air-drying time” are taken as the input and “filter cake moisture” and “processing capacity per unit area” as the output in the OLS and GRNN and SVR method for simulation model construction. The modeling and simulation program is designed in MATLAB language. After running the program, the simulation diagram and accuracy results of the model and test samples are obtained.

When regression model is used for prediction, the common indicators used to analyze and evaluate model errors and accuracy, namely, root mean square error (MSE), mean absolute error (RMAE), and mean relative error (MRE), are included. The MSE refers to the expected value of the square of the difference between the estimated value of the parameter and the true value of the parameter. The MSE can evaluate the degree of fluctuations of the data. The accuracy of the prediction model to describe the experimental data is better when the value of the MSE is smaller. However, the average is generally used. The absolute error is the difference between the measured value (a single measured value or the average of multiple measured values) and the true value, and the relative error is the ratio of the absolute error to the true value. In other words, the credibility of the measurement can be better reflected by the MRE. The calculation formulas of these evaluation indicators are shown in Eqs. (8)–(10).

8 $$RMSE = \sqrt {\frac{1}{m}\sum\limits_{i = 1}^{m} {\left( {y_{i} - \hat{y}_{i} } \right)^{2} } } ,$$

9 $$MRE = \frac{1}{m}\sum\limits_{i = 1}^{m} {\frac{{\left| {y_{i} - \hat{y}_{i} } \right|}}{{y_{i} }}} ,$$

10 $$MAE = \frac{1}{m}\sum\limits_{i = 1}^{m} {\left| {y_{i} - \hat{y}_{i} } \right|} ,$$

where $y_{i}$ is the true value, $\hat{y}_{i}$ is the estimated value or simulation value, and $m$ is the number of test sample.

Table 2 shows that the SVR method has the lowest MRE for the simulation of moisture and processing capacity. In general, the simulation errors are relatively considerable. We mainly consider the complexity and difficulty of data collection, as well as the small number of samples we have temporarily obtained. Thus, a large error is generated in the entire experimental simulation, which inevitably leads to unreliable simulation results. Therefore, after the exploration of the laboratory filter press dehydration process modeling method is completed, the production data directly collected from the industry are used to conduct the modeling and optimization research of the industrial filter press dehydration process.

**Table 2:** Laboratory simulation results of three ML models.

Simulation parameters	ML model	MRE/%	MSE/%	MAE
Simulation of moisture	RBF–OLS	7.12	6.65	0.41
	RBF–GRNN	7.62	3.24	0.46
	**SVR**	**5.85**	**0.95**	**0.37**
Simulation of processing capacity	RBF–OLS	12.65	10.65	39.25
	RBF–GRNN	15.35	7.63	45.12
	**SVR**	**5.36**	**1.35**	**37.78**

Significant values are given in bold.

## Optimal result of filter press dehydration process control in industry

The experimental simulation results of the model of laboratory filter press dehydration process control show that the accuracy of the simulation results of the three ML models with a small sample of experimental data is too small. Considering that the experimental simulation data samples are less, and the laboratory simulation model uses a self-developed micro-automatic filter press dehydration device, the established model is very different from the industrial filter press dehydration control model, the overall experimental simulation accuracy is low. Therefore, we would like to build a better ML model by appropriately adding some actual industrial production data samples. This way can also achieve good results in the actual industrial filter press dehydration process control, which is our goal.

### Industrial simulation results of three ML models

**Figure 9:**
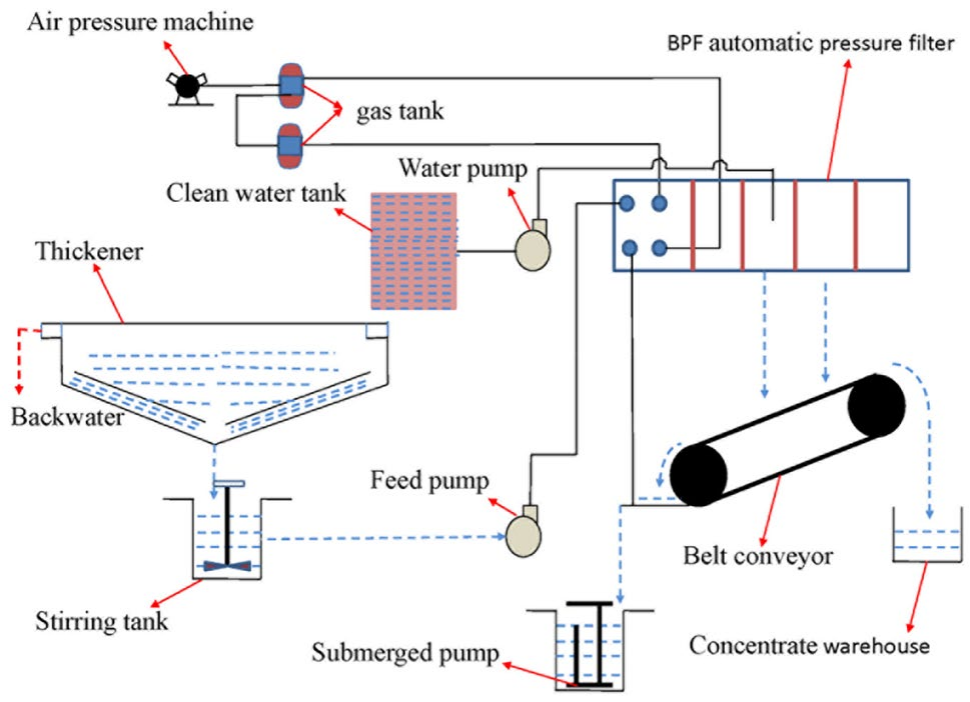
Dewatering system for industrial filter press.

Research is conducted by modeling and optimizing the industrial filter press dehydration system of the flotation gold concentrate of the Miaoling Gold Mine. The BPF automatic filter press is used in the system. The dehydration system is shown in Fig. 9.

We collect data samples from the flotation gold concentrate of the Miaoling Gold Mine in Henan, China for 7 working days and collect 161 sets of industrial data. The size of the gold concentrate used is < 0.074 mm, which accounts for 75% of the total, and the grade is 30 g/t (AU). The first 100 sets of data are used as training samples to construct the simulation model of the filter press dehydration process. Then, the last 61 sets of data are used as test samples to verify the simulation result of the constructed model.

In the modeling and simulation using the OLS and GRNN and SVR method, we take “feeding concentration”, “feeding time”, “squeezing time”, and “air-drying time” as the input and “filter cake moisture” and “processing capacity per unit area” as the output. MATLAB language is used for programming. The MRE values of the three methods for the simulation of test samples are shown in Table 3.

**Table 3:** Industrial simulation results of three ML models.

Simulation parameters	ML model	MRE/%
Simulation of moisture	RBF–OLS	4.92
	RBF–GRNN	4.68
	**SVR**	**1.57**
Simulation of processing capacity	RBF–OLS	8.19
	RBF–GRNN	7.12
	**SVR**	**3.81**

Significant values are given in bold.

**Figure 10:**
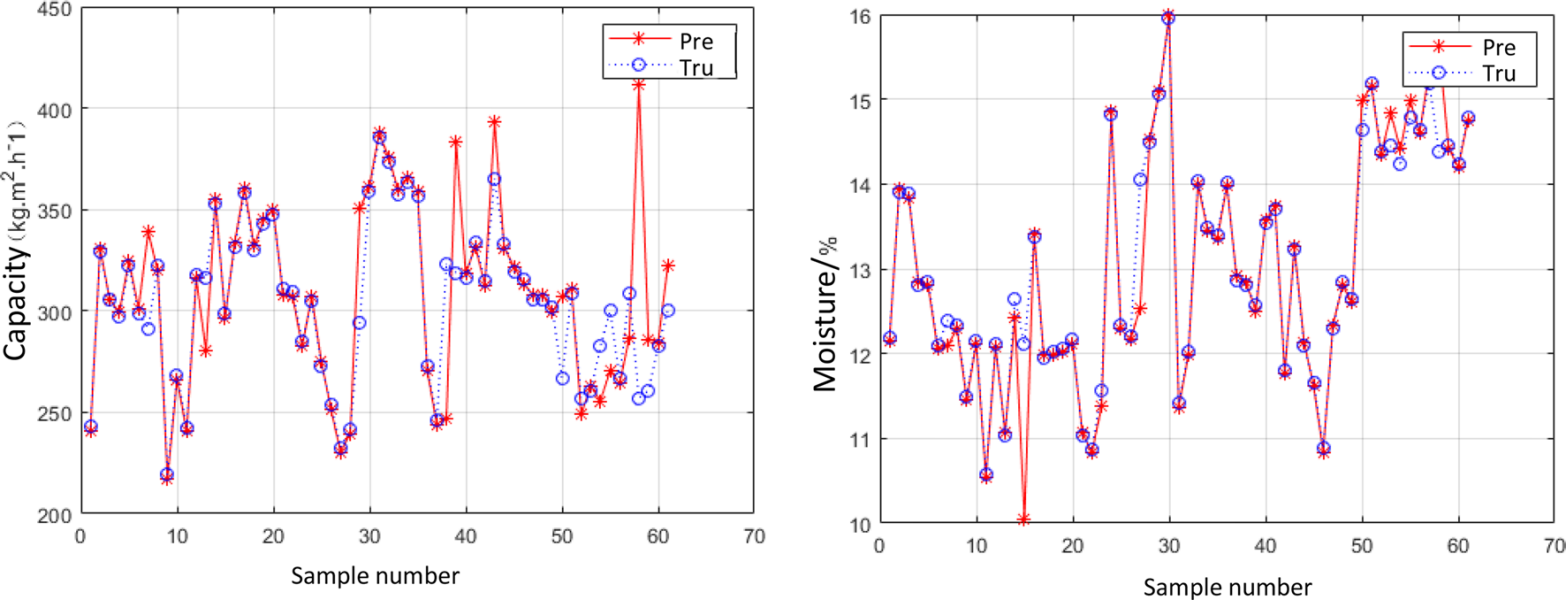
Simulation results of industrial test samples.

Table 3 shows that the model constructed by the SVR method has the highest simulation accuracy and generalization performance. The simulation results of the SVR model on the test sample are shown in Fig. 10. The value has a good approximation to the actual industrial data value. The simulation accuracy of the SVR model for the industrial filter press dehydration process is 98.43%, and the simulation accuracy of the industrial filter press dehydration process is 96.19%. Therefore, the combination of the SVR model and the optimization method based on the control parameters of the filter press dehydration process is utilized in the following analysis. As shown in Eq. (11), the accuracy what we call is relative to MRE.

11 $$Accuracy = 1 - \frac{1}{m}\sum\limits_{i = 1}^{m} {\frac{{\left| {y_{i} - \hat{y}_{i} } \right|}}{{y_{i} }}} .$$

### Test of industrial model by new data

Because the GIGO (garbage in, garbage out) potential of the ML techniques, even the best algorithm will not be helpful if the data quality is poor. Therefore, before considering the reliability of model, more data should be obtained to conduct new test. We reacquire 30 sets of data from the system of filter press dehydration in Fig. 9. Before each filter cake is obtained, we record the corresponding parameters of the system, such as feeding concentration, air-drying time, feeding time, and pressing time. We use these parameters as new inputs, import them into the trained industrial model, and obtain the output predicted by the model: the moisture (%) of filter cake and processing capacity (kg/m^2^/h) unit area per hour.

**Figure 11:**
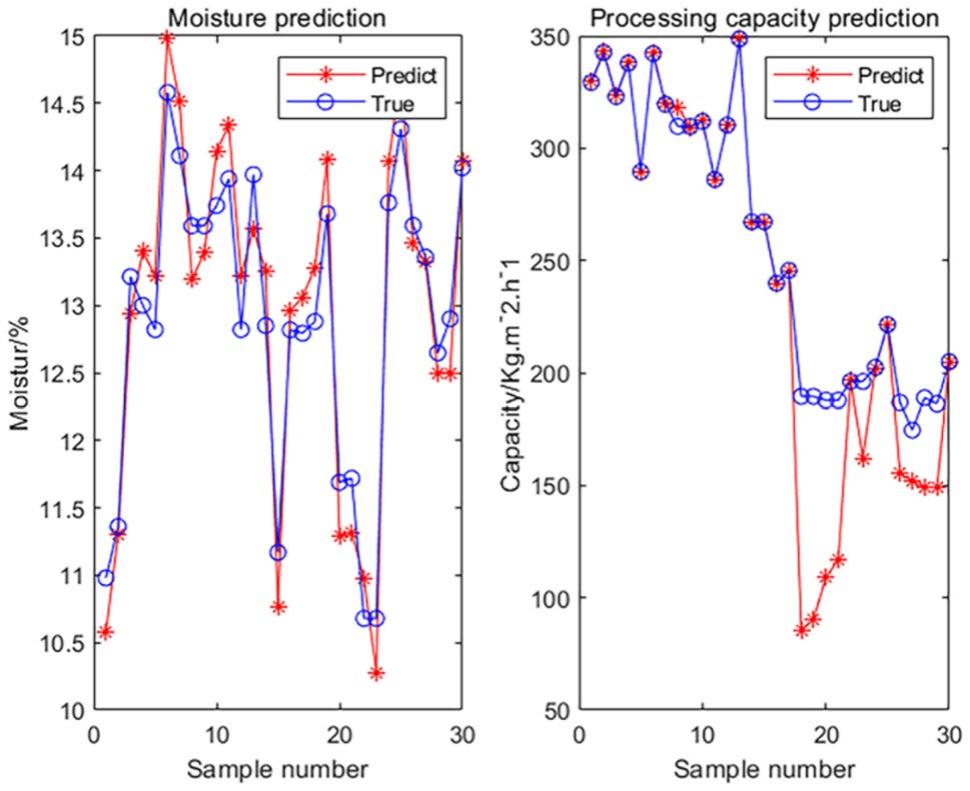
Results of new data prediction.

To ensure the stability and reliability of the data, the 30 groups of data are divided into 15 days, and only 2 groups are obtained every day. We screen, sample, and air-dry the obtained filter cake. The water content and the value of the processing capacity per unit area per hour of the filter cake are calculated, and they are used as the true value of the output. The output value predicted by the new data is compared with the true value of the output, as shown in Fig. 11. The errors between the predicted and true output values is low, which shows that the models and data prediction trends we have obtained from the previous data training have considerable agreement.

### Industrial results of parameter optimization based on control variables

“Method of filter press dehydration optimization” section introduces the optimization method of the filter press dehydration process, and it adopts the optimization method based on control variables. In Chapter 3, we introduce laboratory simulations of three ML models. In “Industrial simulation results of three ML models” section, we introduce the three machine models in the industrial experiment simulation. We adopt the SVR simulation model as the optimization model for the final filter press dehydration process control. After the model is constructed, we can optimize the control parameters of industrial filter press dehydration process accordingly. Therefore, in this section, we focus on the actual results of the optimization method based on the principle of controlled variables in the industry. The optimization program is designed in MATLAB language. After running the program, the optimal control parameter table of each condition and predicted value is obtained.

Considering that the moisture value and the processing capacity are a pair of contradictory indicators, the two indicators cannot be optimal at the same time. We first control the expected moisture value to 12%, that is, the moisture value in the industrial filter press dehydration process is required to be 12% or less to be qualified. Then, the value (12% moisture value) is controlled and constrained in the specific industrial experimental model simulation. Finally, we can obtain another corresponding simulation output value (processing capacity). After the processing capacity value is sorted in ascending order, we obtain the top four processing capacity values. Group data are shown in Table 4. We find through the optimization method based on control variables that the optimal control parameter group in the industrial filter press dehydration process is the data group No. 4*.*

**Table 4:** Results of the optimization method based on the principle of controlled variables in the industry.

Number	Feeding concentration/%	Feeding time/s	Squeezing time/s	Air-drying time/s	Cake moisture/%	Processing capacity (kg/m^2^/h)
1	40	35	15	90	**12.0**	280.3
2	45	40	20	85	**12.0**	304.2
3	50	35	35	75	**12.0**	325.8
**4**	**55**	**35**	**35**	**85**	**12.0**	**347.5**

Significant values are given in bold.

**Figure 12:**
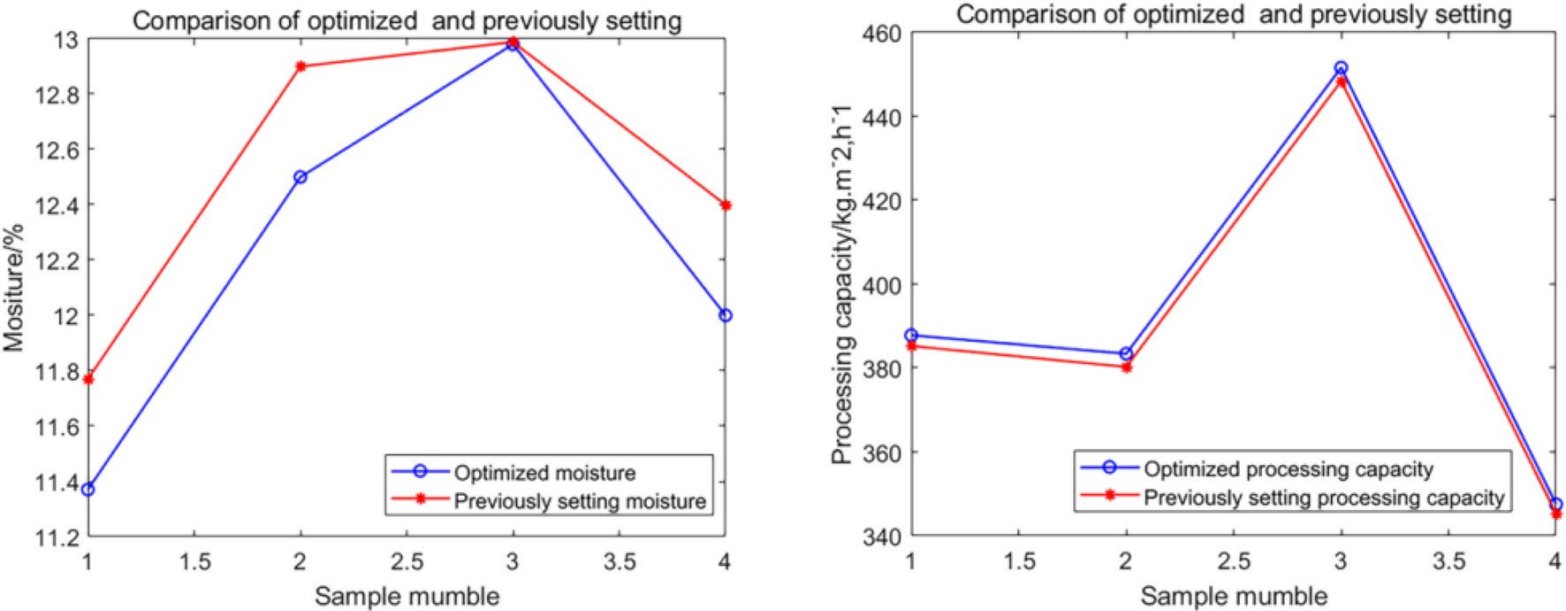
Validation of the optimized condition.

In order to verify the optimized condition by comparing results showing the filter operating under the previous setting and with the optimized setting, we obtained four sets of moisture value and processing capacity from the filter press system before and after optimization, as shown in Fig. 12 and Table 5. Optimized moisture and processing capacity obtained a better optimization effect than previously setting moisture and processing capacity, which is enough to prove that this optimization method can continuously optimize and improve the dehydration index.

**Table 5:** Comparison of optimized indicators and previously setting indicators.

Number	Optimized moisture/%	Previously setting moisture/%	Improved dehydration performance/%	Optimized processing capacity (kg/m^2^/h)	Previously setting processing capacity (kg/m^2^/h)	Increased processing capacity/%
1	11.3	11.8	4.42	387.8	385.3	0.91
2	12.3	12.6	2.44	383.4	380.2	0.84
3	12.7	13.1	3.15	451.6	448.4	0.71
4	11.0	12.4	12.73	347.5	345.3	0.63

### Industrial application of filter press dehydration optimization control

**Figure 13:**
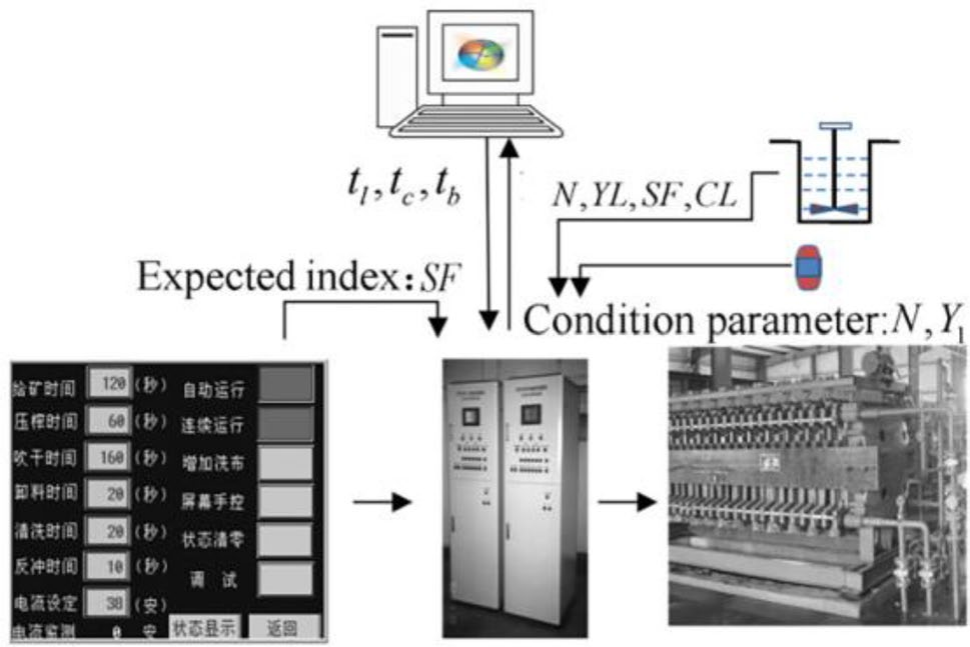
Adaptive control system of filter press dehydration process.

After the optimization results of the control parameters are obtained, the optimal control parameters can be directly obtained according to the condition parameters and optimization goals. This way guides the setting and adjustment of the control parameters of the dehydration process and realizes the optimization of the process. Simultaneously, the optimization results obtained in this study are used as training samples, and a SVR simulation model with optimized parameters is constructed. This model can be used to achieve adaptive optimization control of the filter press dehydration process. The system configuration is shown in Fig. 13. In actual industrial applications, we first set the expected index of the filter press dehydration process through the computer. Then, we collect the working condition parameters through the computer in real time to obtain the corresponding work index. Finally, a computer is used to compare the degree of gap between the work and expected indicators. The various parameters of the filter press are adjusted in real time until they are the closest to the expected index. In this way, adaptive control of the filter press dehydration process can be realized.

Industrial practice has proven that the proposed optimization method not only can ensure the stability of the filter cake moisture content but also can shorten the filter press operation cycle to less than 85% of the original. This way reduces the single-shift operation time of the filter press system, correspondingly saves production energy consumption and cost, and improves the efficiency of filter press operation.

## Conclusions

A large number of dehydration process optimization studies have given us many exciting ideas and have made certain contributions to industrial production practice. In this study, we summarize the existing research on the optimization of dehydration process, and recognize the insufficiency of intelligence in the dehydration process and the limited optimization effect. Therefore, a laboratory filter press dehydration system and an industrial filter press dehydration system were built, and multiple sets of data were collected. Three machine learning models of RBF–OLS, RBF–GRNN and SVR were constructed, combined with an optimization method based on control variables. Specifically, the following work has been done:

1. Laboratory experiments and industrial experiments have done. In the laboratory, a self-developed micro-automatic filter press for data sampling is used, in industry, the data is obtained directly from the large-scale filter press dehydration equipment of the industrial dehydration system. It is found that simulation results of the SVR in the laboratory are relatively poor, but SVR shows excellent performance in the industrial simulation results.
2. In order to further verify the reliability of the industrial model of concentrate filter press dehydration, we once again obtained the corresponding data from the industrial filter press dehydration system to predict the indicators. By comparing the indicators predicted by the model with the actual indicators, it is found that the percentage error between them is considerable, the optimal control parameter combination of the industrial filter press dehydration process is successfully obtained using the method of optimizing the control parameters of the filter press dehydration process by the control variables. Practice has proven that guiding the production with the obtained optimal control parameter combination not only can ensure the stability of the filter press production index but also can reduce energy consumption and significantly improves the efficiency of filter press operations.
3. In order to verify the rationality of the optimization method based on the control variable method, under the previous setting and with the optimized setting, the indicators of the filter cake moisture and the unit area processing capacity per hour in the industrial filter press dehydration system are respectively compared. Experiments have proved that Through the machine learning model combined with the optimization method based on control variables, the continuous optimization of the dehydration process can be achieved, the optimization ability can be guaranteed, the production indicators can be continuously optimized, the production process can be guided, and the difficulty of continuous parameter adjustment by technical workers can be alleviated, initial prototype of intelligence is formed.
